# Supplementary material for: Halenaquinol Blocks Staphylococcal Protein A Anchoring on Cell Wall Surface by Inhibiting Sortase A in Staphylococcus aureus
Source: Mar Drugs. 2024 Jun 10;22(6):266. doi: 10.3390/md22060266 (PMC11204543; doi:10.3390/md22060266)
Supplement: Supplementary file 1 [file marinedrugs-22-00266-s001.zip › marinedrugs-3042541-supplementary.pdf]

Supporting Information

Halenaquinol Blocks Staphylococcal Protein A Anchoring on Cell Wall Surface by Inhibiting Sortase A in *Staphylococcus aureus*

Jaepil Lee [1], Jae-Hyeong Choi[2,3], Jayho Lee [1], Eunji Cho [1], Yeon-Ju Lee[2,3], Hyi-Seung Lee [2,3,*] and Ki-Bong Oh [1,*]

[1] *Department of Agricultural Biotechnology, College of Agriculture and Life Sciences and Natural Products Research Institute, Seoul National University, Seoul 08826, Republic of Korea*

[2] *Marine Natural Products Chemistry Laboratory, Korea Institute of Ocean Science and Technology, Busan 49111, Republic of Korea*

[3] *Department of Applied Ocean Science, University of Science and Technology, Daejeon 34113, Republic of Korea*

Contents

Figure S1. ^{1}H NMR spectrum in CD_3OD of compound **1**.

Figure S2. ^{13}C NMR spectrum in CD_3OD of compound **1**.

Figure S3. ^{1}H NMR spectrum in DMSO-d_6 of compound **2**.

Figure S4. ^{13}C NMR spectrum in DMSO-d_6 of compound **2**.

Figure S5. ^{1}H NMR spectrum in CD_3OD of compound **3**.

Figure S6. ^{13}C NMR spectrum in CD_3OD of compound **3**.

Figure S7. ^{1}H NMR spectrum in CD_3OD of compound **4**.

Figure S8. ^{13}C NMR spectrum in CD_3OD of compound **4**.

Figure S9. ^{1}H NMR spectrum in DMSO-d_6 of compound **5**.

Figure S10. ^{13}C NMR spectrum in DMSO-d_6 of compound **5**.

Figure S11. ^{1}H NMR spectrum in DMSO-d_6 of compound **6**.

Figure S12. ^{13}C NMR spectrum in DMSO-d_6 of compound **6**.

‘

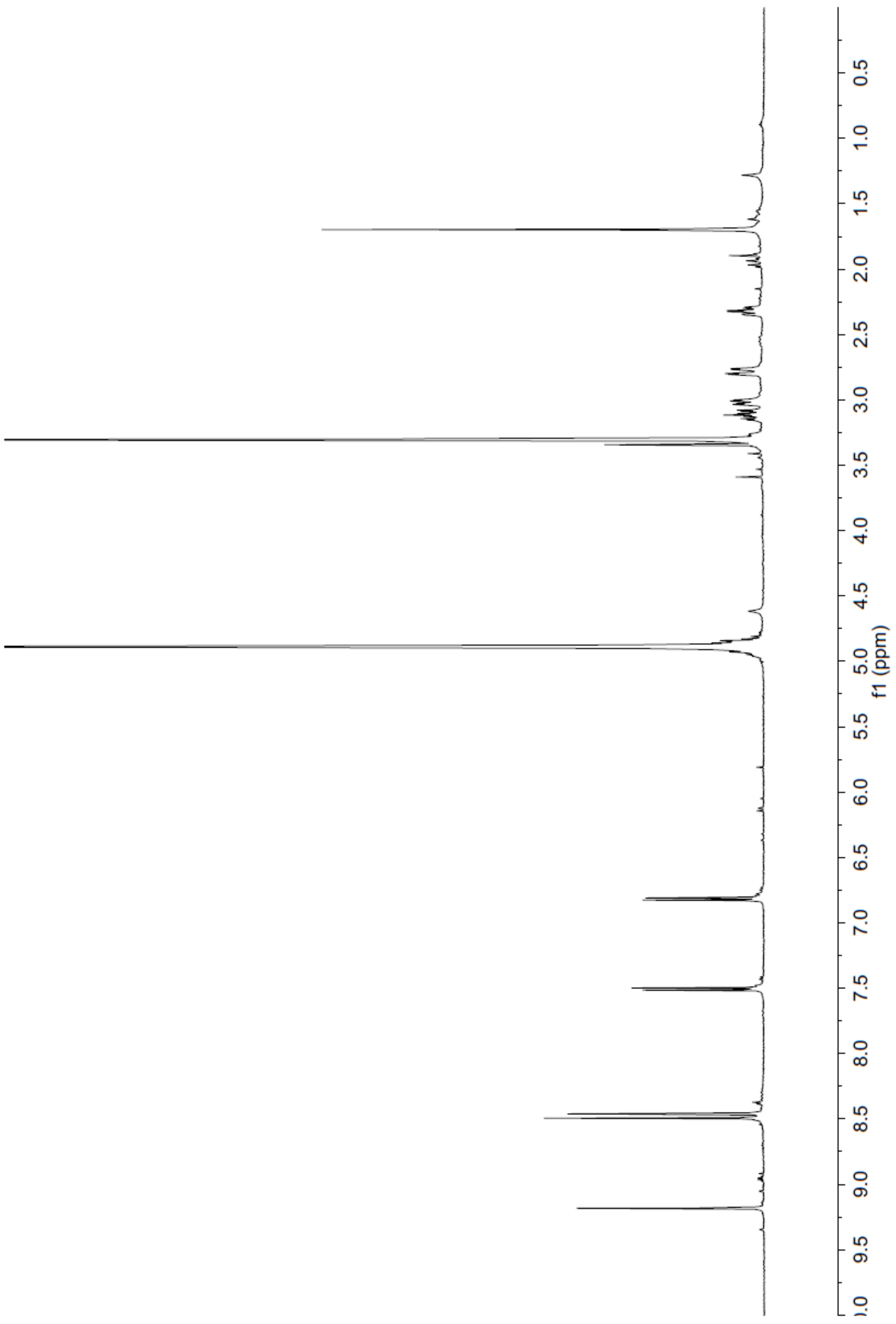

Figure S1. ^{1}H NMR spectrum in CD_3OD of compound **1**.

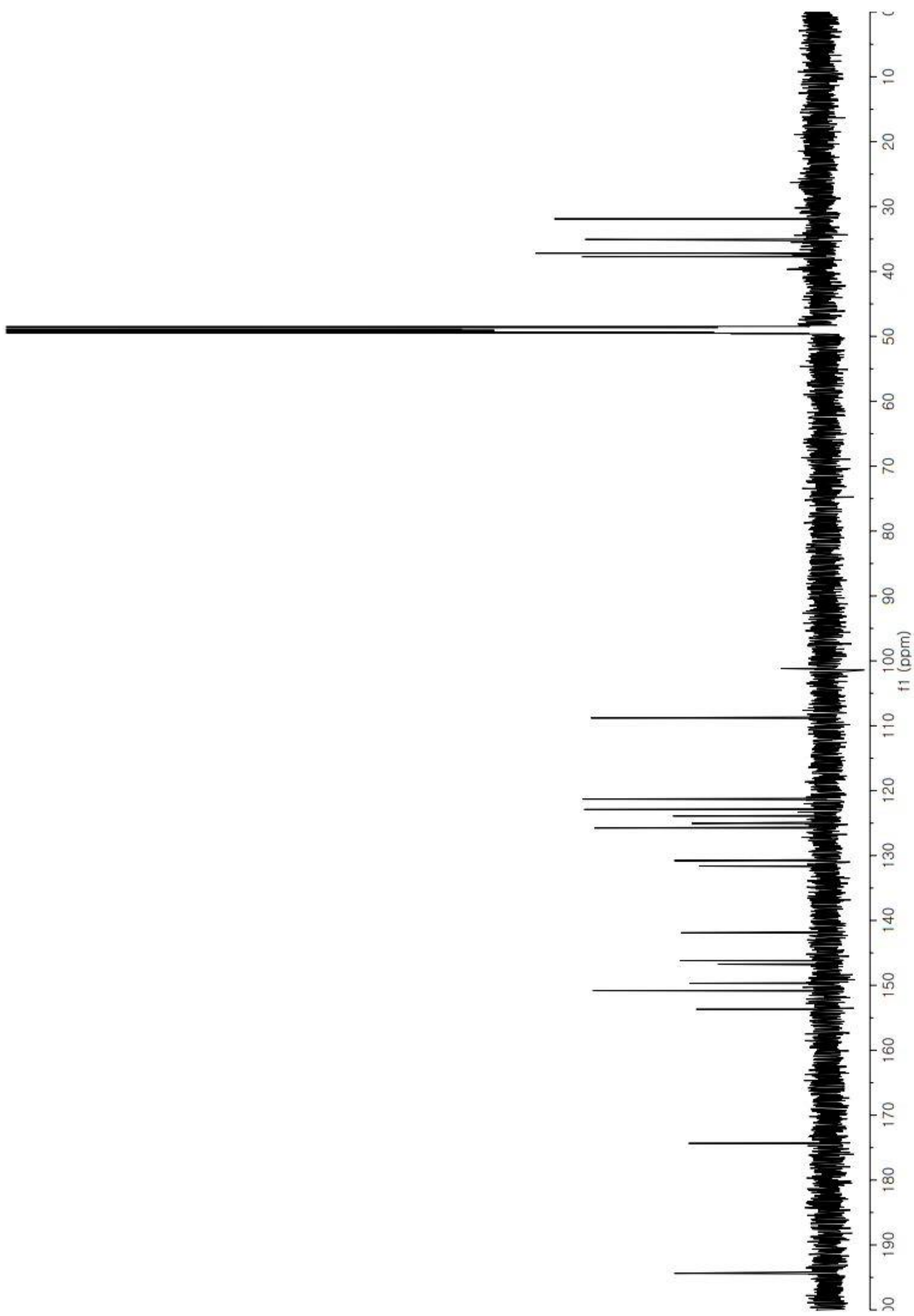

Figure S2. ^{13}C NMR spectrum in CD_3OD of compound **1**.

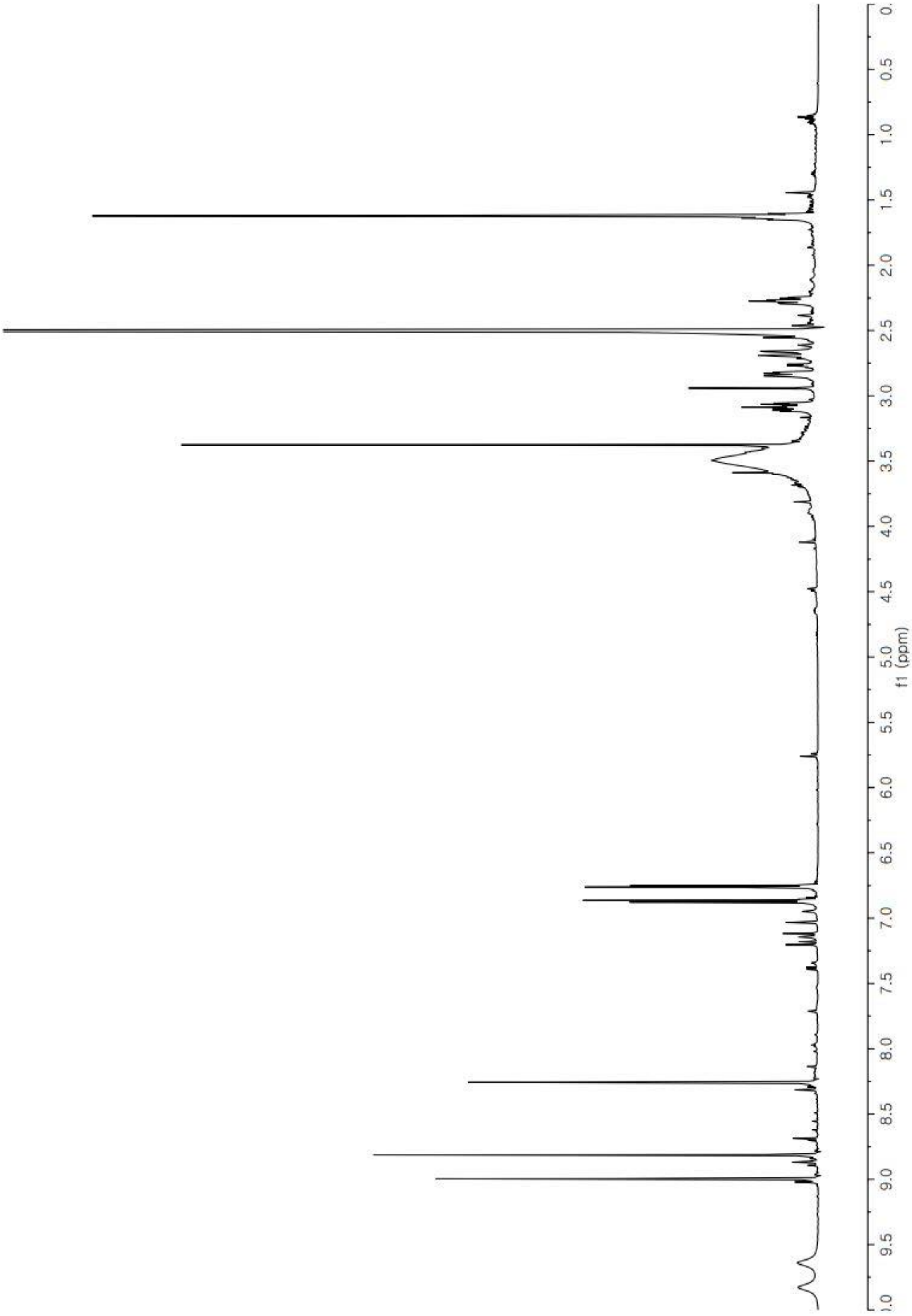

Figure S3. ^{1}H NMR spectrum in DMSO-d_6 of compound **2**.

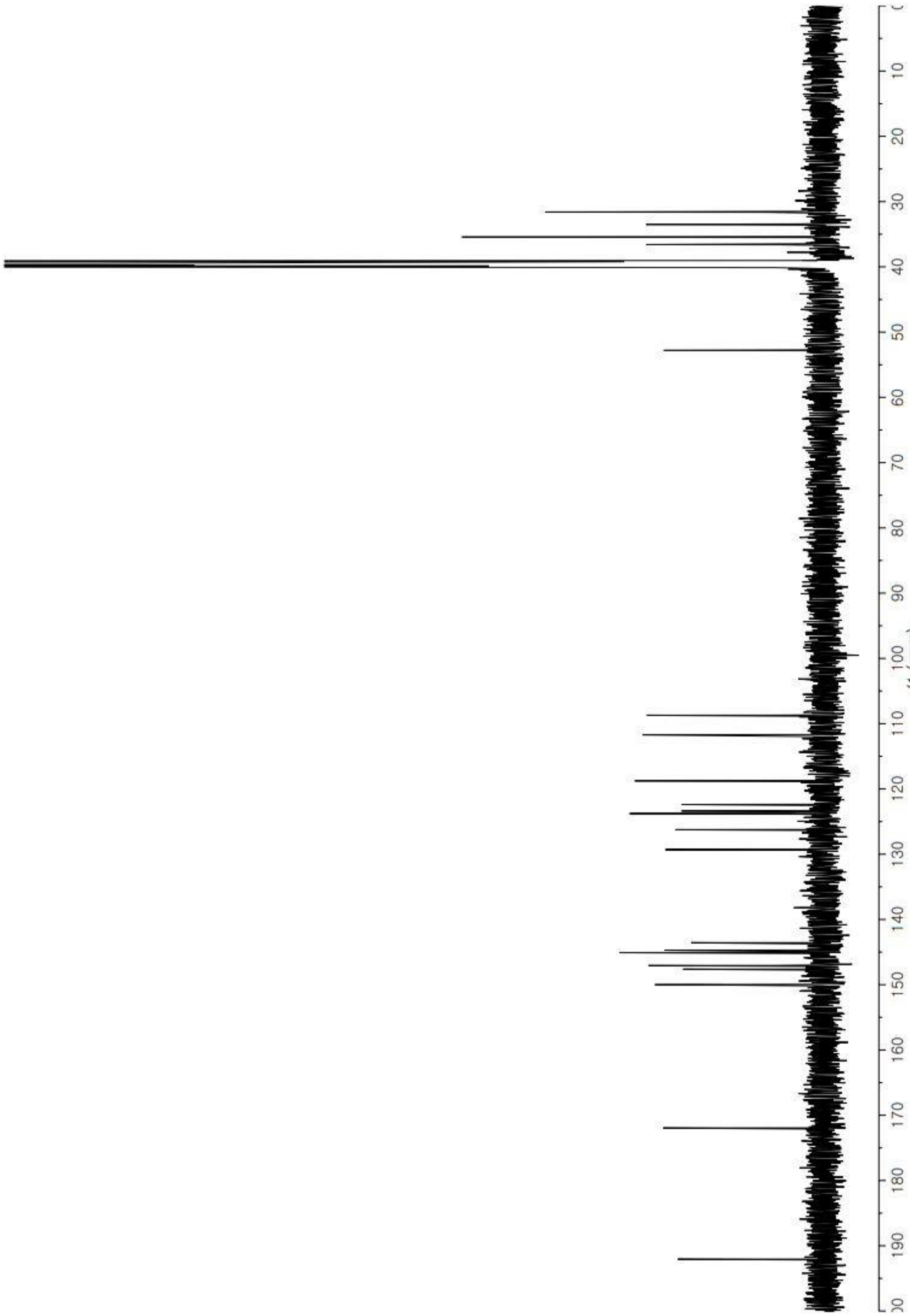

Figure S4. ^{13}C NMR spectrum in DMSO-d_6 of compound **2**.

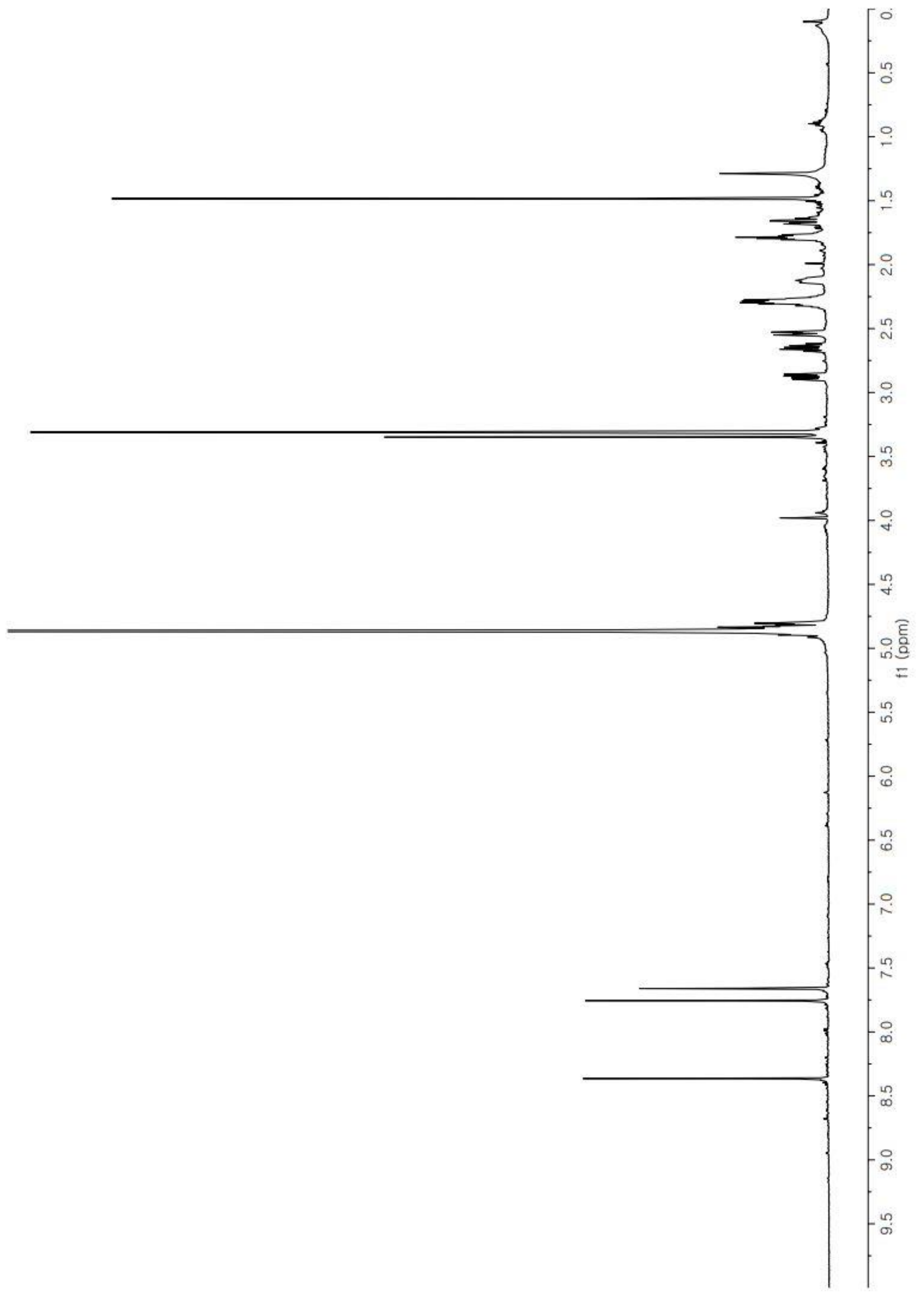

Figure S5. ^{1}H NMR spectrum in CD_3OD of compound **3**.

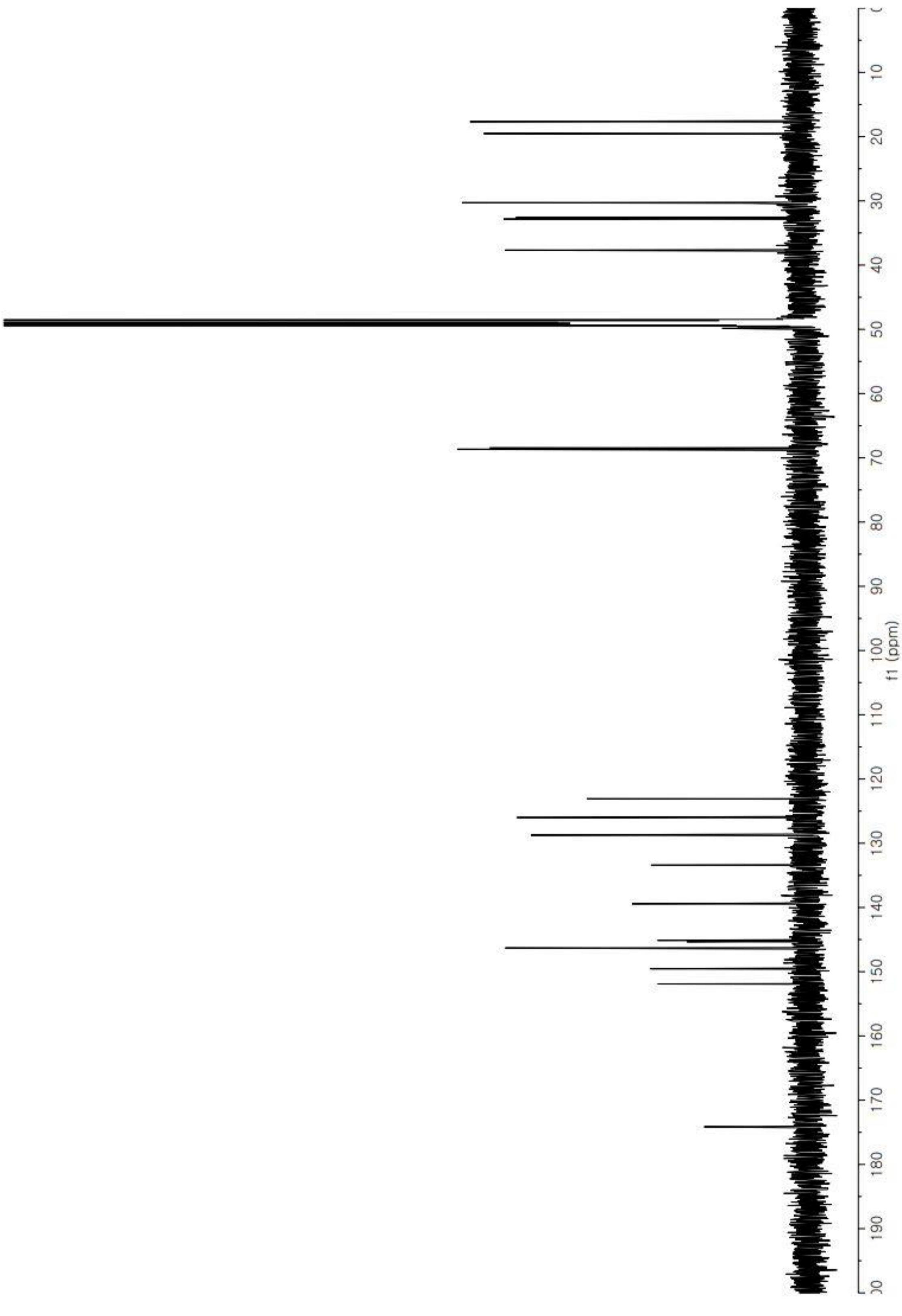

Figure S6. ^{13}C NMR spectrum in CD_3OD of compound **3**.

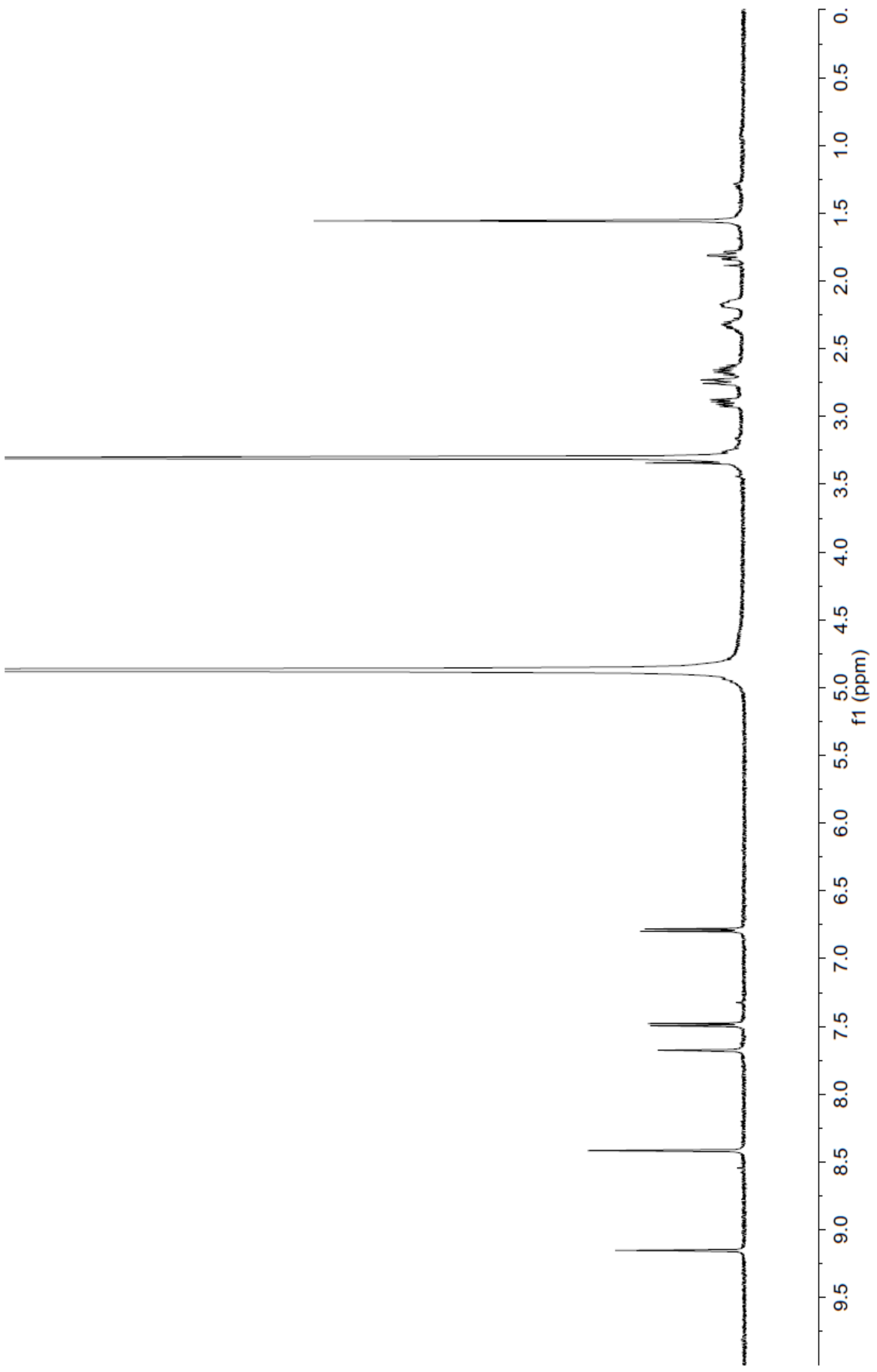

Figure S7. ^{1}H NMR spectrum in CD_3OD of compound **4**.

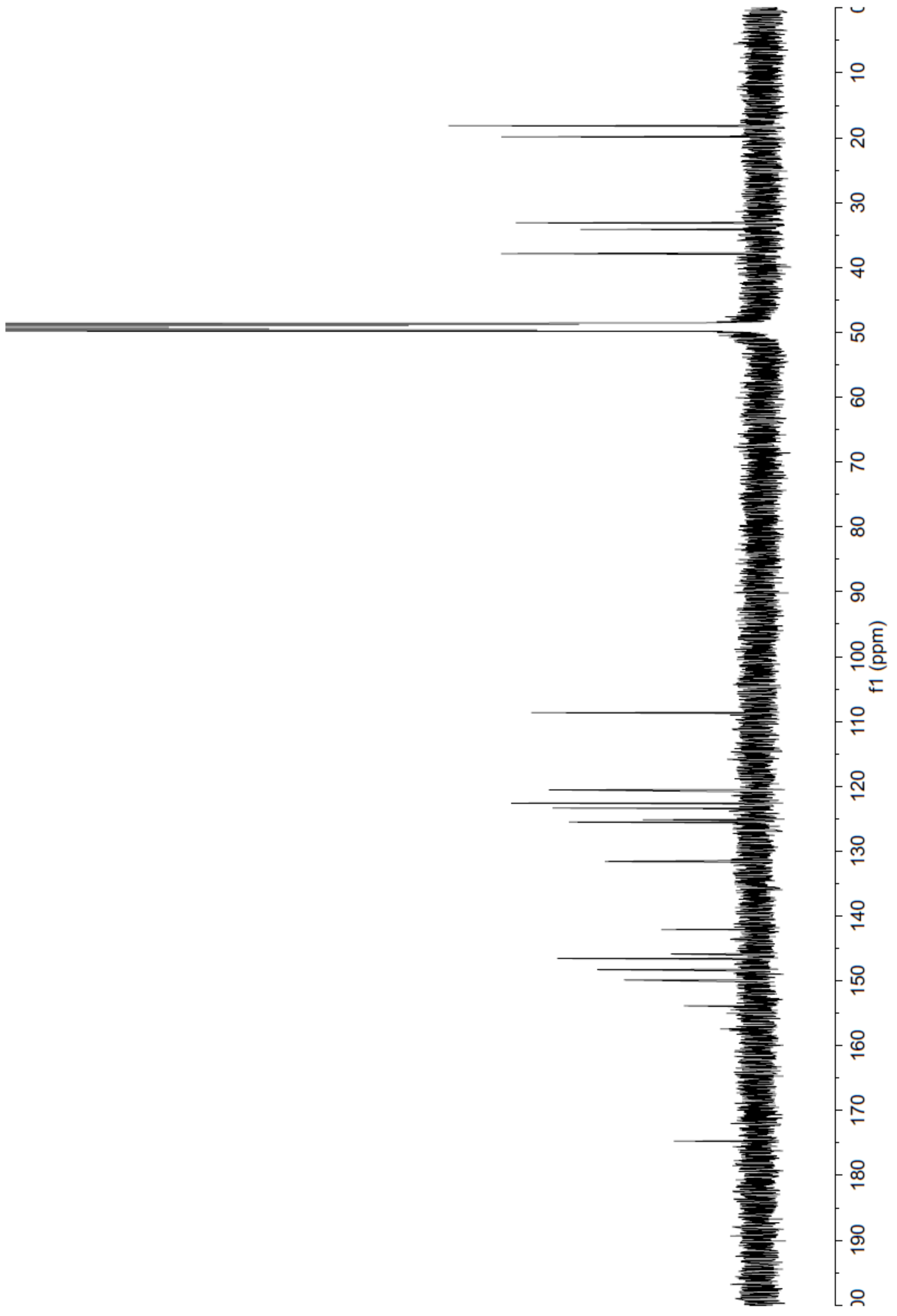

Figure S8. ^{13}C NMR spectrum in CD_3OD of compound **4**.

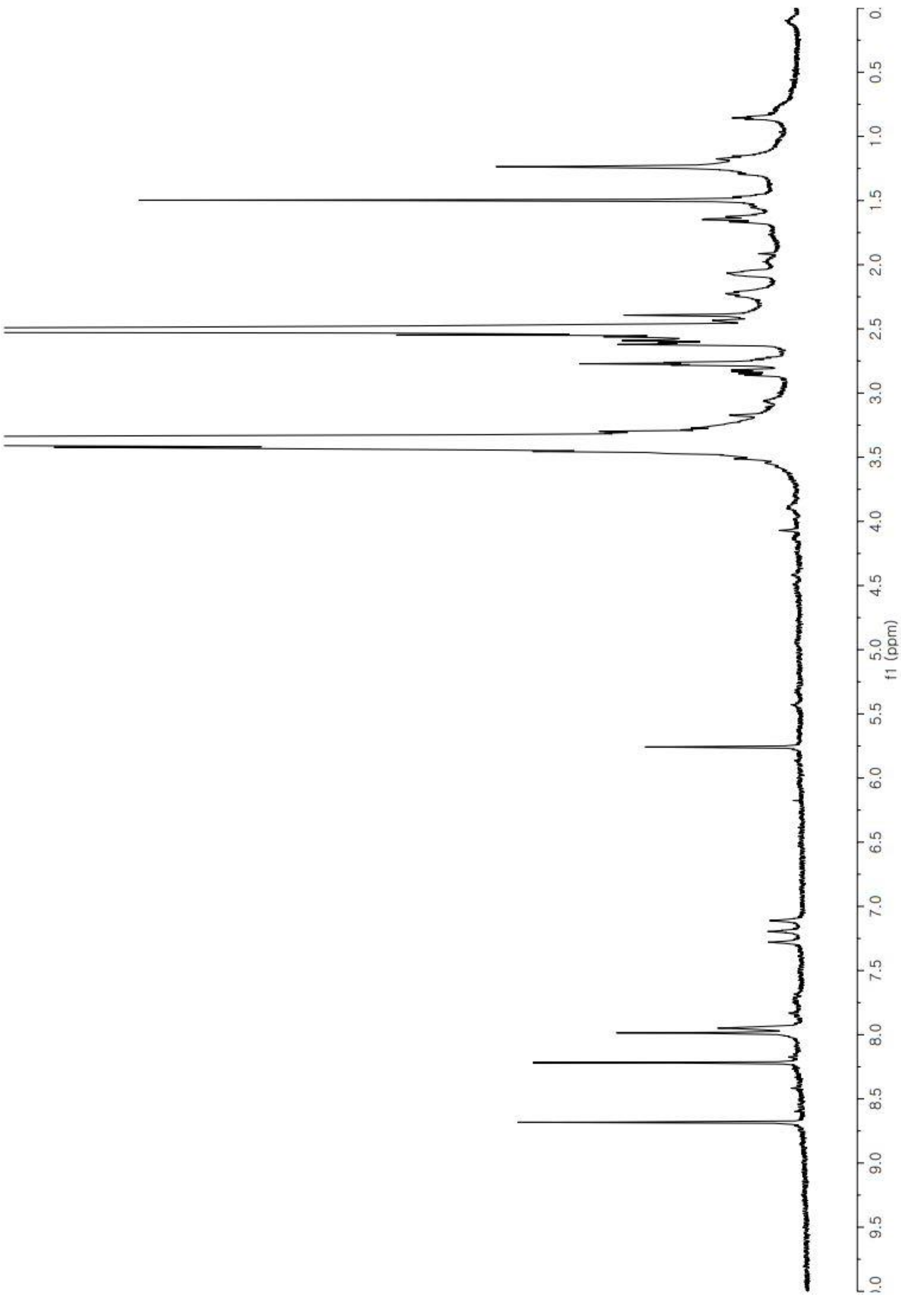

Figure S9. ^1H NMR spectrum in DMSO-d_6 of compound 5.

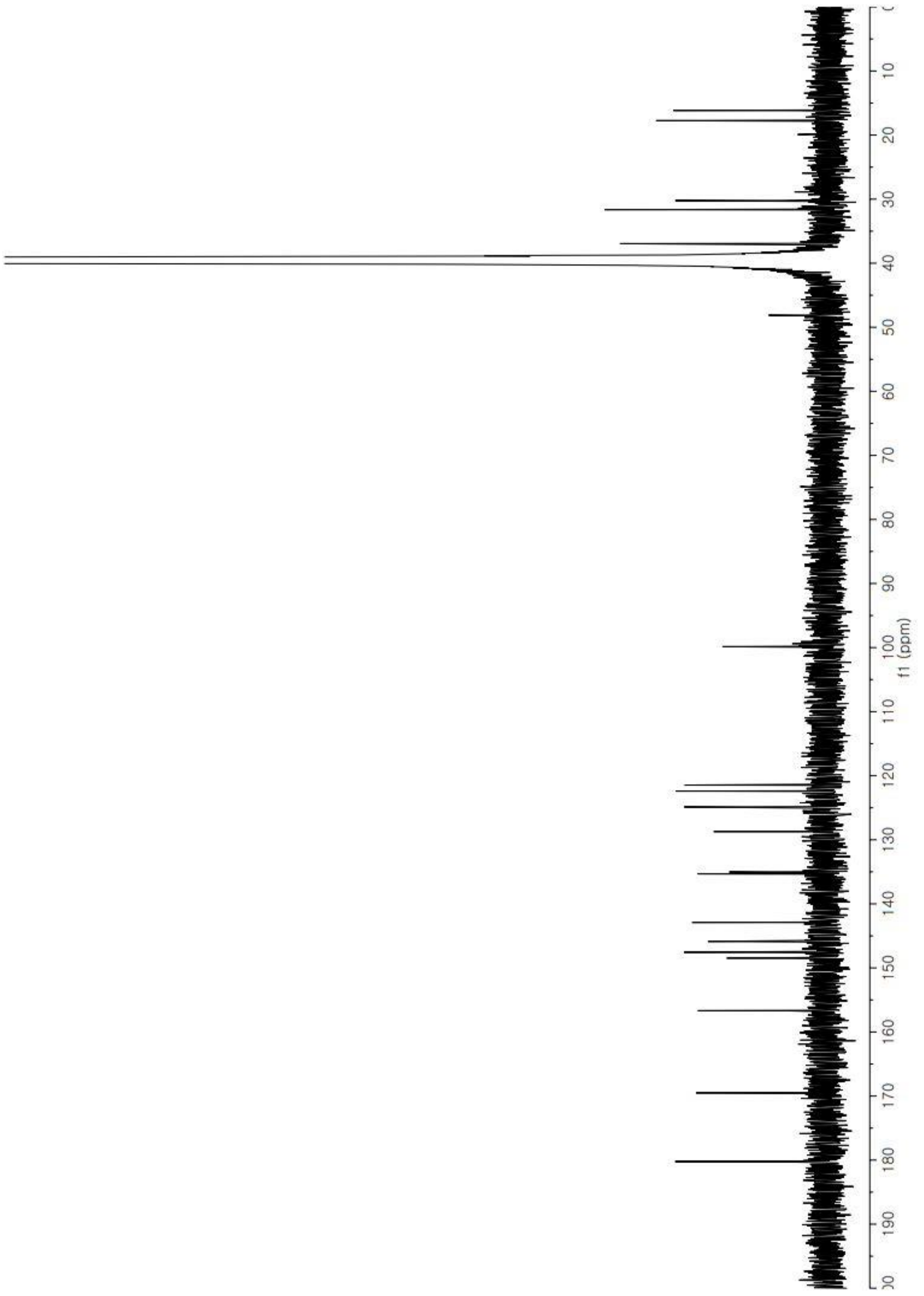

Figure S10. ^{13}C NMR spectrum in DMSO-d_6 of compound **5**.

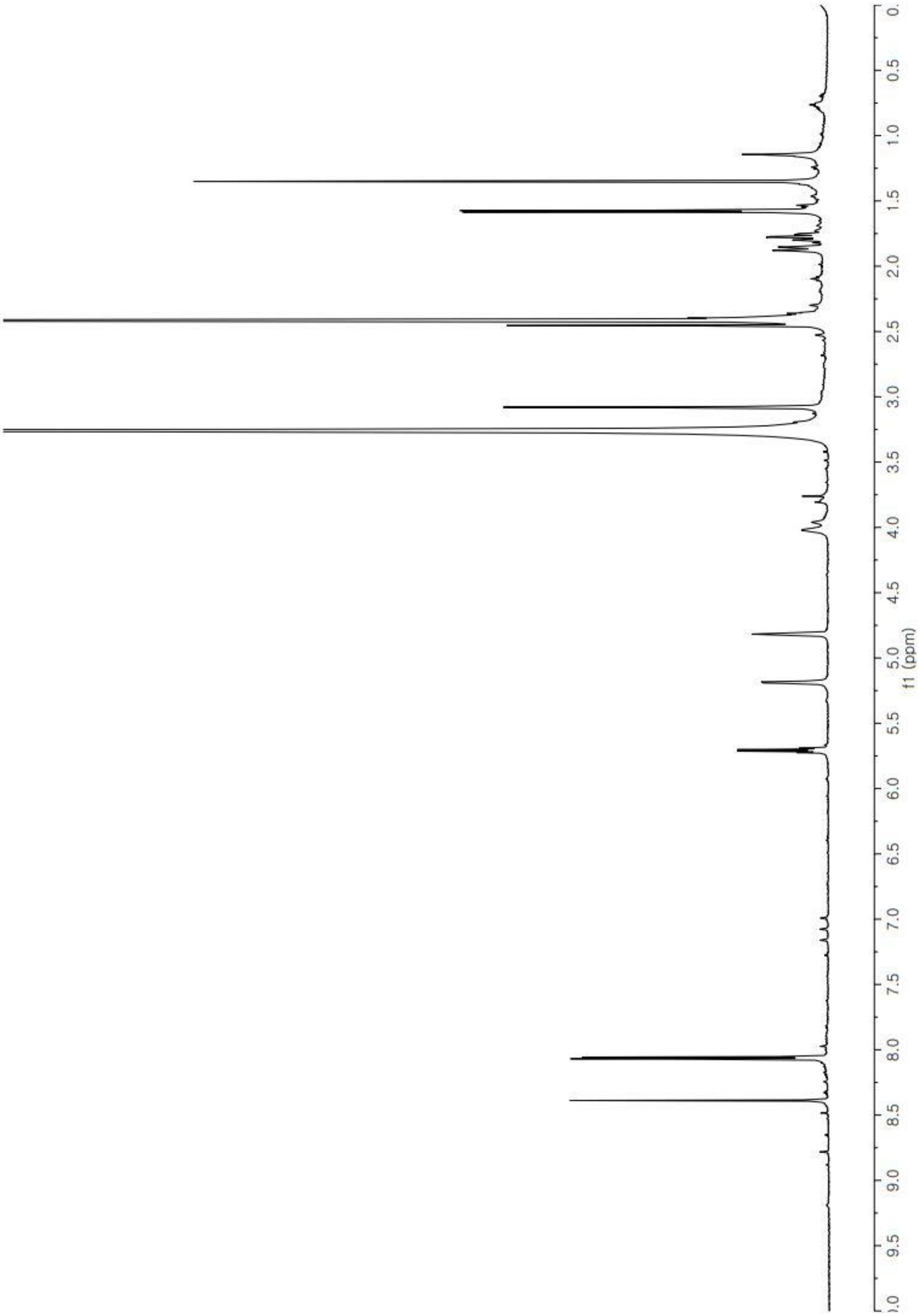

Figure S11. ^{1}H NMR spectrum in DMSO-d_6 of compound **6**.

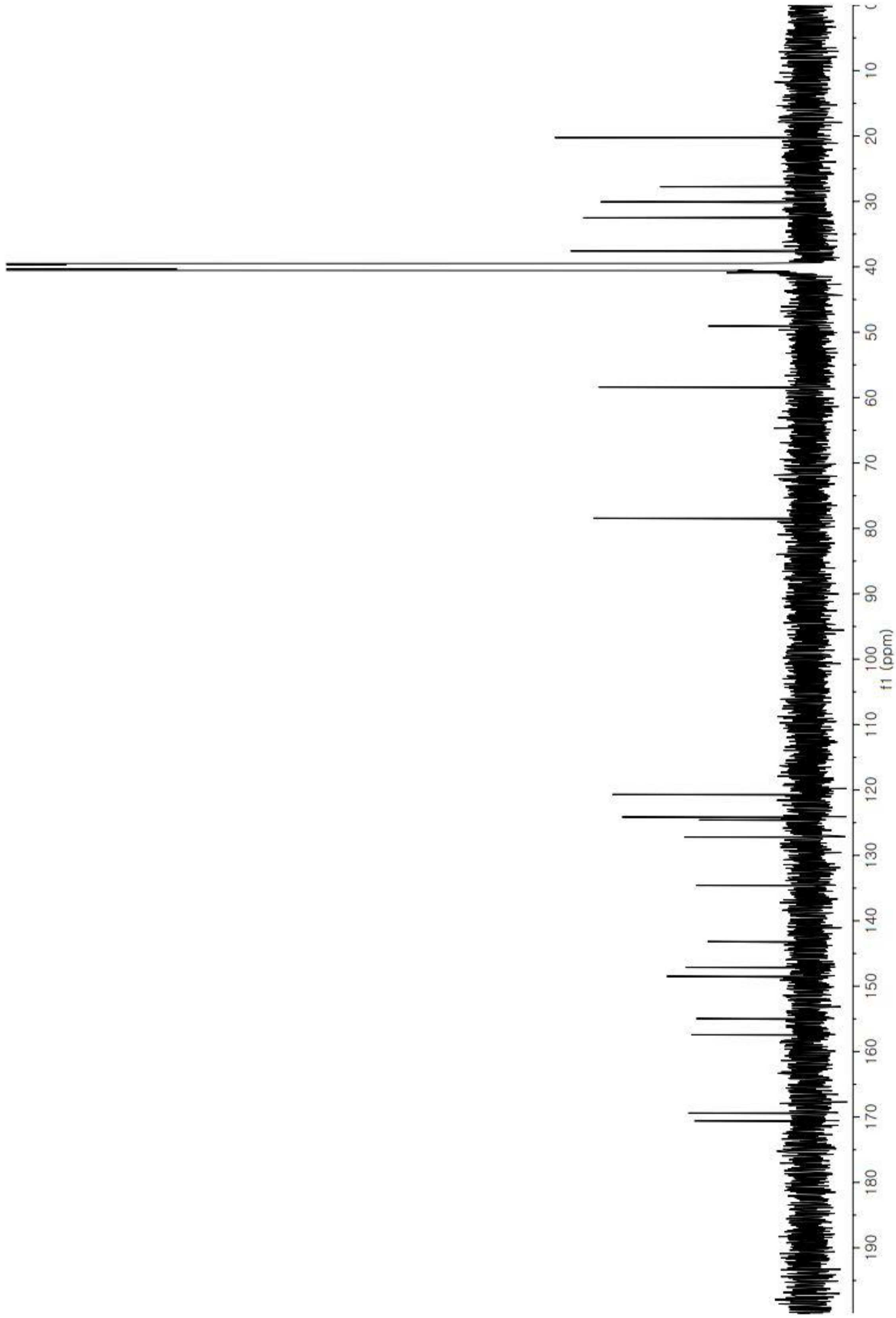

Figure S12. ^{13}C NMR spectrum in DMSO-d_6 of compound **6**.
